# Cardiovascular complications in newly diagnosed rheumatic heart disease patients at Mulago Hospital, Uganda

**DOI:** 10.5830/CVJA-2013-004

**Published:** 2013-04

**Authors:** Emmy Okello, Zhang Wanzhu, Charles Musoke, Barbara Kakande, Charles K Mondo, Juergen Freers, Aliku Twalib, Peter Lwabi, Nyakoojo B Wilson, R Odoi-Adome

**Affiliations:** Department of Medicine, Division of Cardiology, College of Health Sciences, Makerere University, Uganda; Uganda Heart Institute, Mulago National Referral Hospital, Makerere University, Uganda; Department of Medicine, Division of Cardiology, College of Health Sciences, Makerere University, Uganda; Department of Medicine, Division of Cardiology, College of Health Sciences, Makerere University, Uganda; Department of Medicine, Division of Cardiology, College of Health Sciences, Makerere University, Uganda; Uganda Heart Institute, Mulago National Referral Hospital, Makerere University, Uganda; Department of Medicine, Division of Cardiology, College of Health Sciences, Makerere University, Uganda; Uganda Heart Institute, Mulago National Referral Hospital, Makerere University, Uganda; Department of Medicine, Division of Cardiology, College of Health Sciences, Makerere University, Uganda; Uganda Heart Institute, Mulago National Referral Hospital, Makerere University, Uganda; Uganda Heart Institute, Mulago National Referral Hospital, Makerere University, Uganda; Uganda Heart Institute, Mulago National Referral Hospital, Makerere University, Uganda; School of Pharmacy, Makerere University, Uganda

**Keywords:** rheumatic heart disease, complications, newly diagnosed patients

## Abstract

**Background:**

Complications of rheumatic heart disease are associated with severe morbidity and mortality in developing countries where the disease prevalence remains high. Due to lack of screening services, many patients present late, with severe valve disease. In Uganda, the disease and its complications are still not well studied.

**Objective:**

To profile and describe cardiovascular complications in newly diagnosed rheumatic heart disease patients attending the Mulago National Referral Hospital in Uganda.

**Methods:**

This was a cross-sectional study where consecutive, newly diagnosed rheumatic heart disease patients were assessed and followed up for complications, such as heart failure, pulmonary hypertension, atrial fibrillation, recurrence of acute rheumatic fever, and stroke.

**Results:**

A total of 309 (115 males and 196 females) definite rheumatic heart disease patients aged 15–60 years were enrolled in the study and analysed. Complications occurred in 49% (152/309) of the newly diagnosed rheumatic heart disease cases, with heart failure (46.9%) the most common complication, followed by pulmonary arterial hypertension (32.7%), atrial fibrillation (13.9%), recurrence of acute rheumatic fever (11.4%), infective endocarditis (4.5%) and stroke (1.3%). Atrial fibrillation and acute rheumatic fever were the most common complications associated with heart failure.

**Conclusion:**

In this study we found that about 50% of newly diagnosed rheumatic heart disease patients in Uganda presented with complications. Heart failure and pulmonary arterial hypertension were the most commonly observed complications.

## Abstract

Worldwide, rheumatic heart disease (RHD) and its complications result in about 233 000 deaths annually. The World Health Organisation (WHO) estimates that approximately 16 million people are currently affected by the disease.^1^ The majority of RHD cases occur in Africa where prevalence rates are as high as one in 10 people in some communities.^1^-^3^

Acute rheumatic fever is the result of a hyper-immune response to Group A streptococcal infection in the susceptible host. The resulting valvular damage, or RHD, is the only longterm consequence of acute rheumatic fever. Lack of primary prevention (treatment of group A streptococcal infections), and lack of screening programmes to detect early RHD, results in late disease presentation, with most patients only seeking medical care due to symptoms related to complications of the disease. Consequently, patients present with various long-term structural and haemodynamic complications, such as heart failure, atrial fibrillation and stroke.^4^-^6^

The situation is worsened by low clinician knowledge in the diagnosis and treatment of the disease and some of its complications.^7^ Late presentation denies patients an opportunity for early intervention in the management the disease, including early medical treatment and institution of benzathine penicillin prophylaxis, which has been shown to prevent recurrence of acute rheumatic fever.^8^

Apart from a prevalence survey in primary schools, no systemic study has documented the complications of RHD in newly diagnosed patients in Uganda, making it difficult to design guidelines for prevention, diagnosis and treatment. The awareness, surveillance, advocacy and prevention (ASAP) programme was developed to increase awareness and surveillance of RHD in most affected countries.^9^,^10^ The strategy aims at raising awareness among the general public and healthcare workers about RHD, and improving the quality of information regarding RHD. The objective of the present study was to describe complications as found in newly diagnosed RHD patients attending Mulago National Referral Hospital in Uganda.

## Methods

The study was approved by the Institutional Review Board (IRB) of the School of Medicine, Makerere University College of Health Sciences and the Uganda National Council for Science and Technology (UNCST).

Consecutive patients with a diagnosis of RHD were enrolled in this cross-sectional study between June 2010 and January 2012. We report on complication rates at presentation in those who were newly diagnosed with definite RHD.

The study was conducted at Mulago Hospital, Uganda’s main national referral hospital and teaching hospital for the Makerere University College of Health Sciences. Annually, Mulago Hospital admits approximately 60 000 patients and diagnoses approximately 240 new cases of rheumatic heart disease.

For the purposes of this study, we were exclusively interested in patients newly diagnosed with RHD. To be included in this analysis, the patient was required to be between the ages of 15 and 60 years, to have definite RHD (2006 WHO/NIH criteria) confirmed by echocardiography, and to be willing to sign informed consent.

Exclusion criteria included prior diagnosis of RHD, age < 15 or > 60 years, and/or presence of congenital heart disease. Furthermore, patients found to have atrial fibrillation in addition to abnormal electrolyte levels or abnormal thyroid function tests were also excluded. Medical doctors from the Mulago Hospital complex as well as other regional hospitals were invited to participate in the study by referring all patients aged 15 to 60 years with suspected RHD to the study site.

Patients who were referred for evaluation underwent a comprehensive screening evaluation to determine the presence or absence of rheumatic heart disease. A medical history was obtained, including history of acute rheumatic fever (ARF): recent sore throat, joint pain, tremors or skin rash. A comprehensive physical examination including auscultation of the chest was carried out. A specific search for known complications of RHD such as heart failure, pulmonary hypertension, atrial fibrillation, infective endocarditis, stroke and recurrence of acute rheumatic fever was carried out during the physical examination and was later confirmed by specific tests.

Standard transthoracic echocardiography (GE, Vivid 8, Chicago, USA) was preformed according to the American Society of Cardiology guidelines.^11^ Patients found to have congenital heart disease were referred to the Paediatric Cardiology Division for further evaluation. For the remainder of the patients, the 2006 WHO/NIH Joint Consensus Statement on Echocardiographic Diagnosis of RHD was used to classify patients as ‘definite’, ‘probable’, or ‘possible’ RHD, or as ‘no disease’.^12^

Cases confirmed to have definite RHD were asked to sign informed consent. Study participants then completed a detailed demographic profile and clinical questionnaire. They also underwent a chest X-ray and standard 12-lead electrocardiography (Cardiopac, Germany). Finally, 6 ml of venous blood was obtained through peripheral venipuncture, and complete blood counts, anti-streptolysin O (ASO) titres, erythrocyte sedimentation rate and C-reactive protein were determined.

## Echocardiographic definitions

Echocardiographic images were obtained from the parasternal long-axis, parasternal short-axis, apical four- and five-chamber and sub-costal views. Morphological abnormalities of the mitral valve, including thickening or calcification of the leaflets, and fusion, shortening, fibrosis, and /or calcification of the mitral chordae were recorded.

Mitral stenosis was labelled as significant if there was evidence of flow acceleration across the mitral valve with a mean pressure gradient > 4 m/s.^12^ Severity of mitral stenosis was determined by planimetry and pressure half-time methods, as mild (MVA > 1.5 cm^2^), moderate (MVA = 1.1–1.5 cm^2^) and severe (MVA < 1.0 cm^2^). Mitral regurgitation was labelled as significant if it was seen in two views by colour Doppler, was > 2 cm from the coaptation point of the valve leaflets, was high velocity, and persisted throughout systole.^12^ Mitral regurgitation was classified as severe if there was systolic flow reversal in the pulmonary veins.

Morphological abnormalities of the aortic valve, including commissural fusion of the aortic leaflets, increased echogenicity along the leaflet edges, and systolic doming of the aortic leaflets was noted. Aortic stenosis was graded based on valve area as well as using flow velocity and mean pressure gradient across the valve (mild if valve area > 1.5 cm, moderate if valve area was 1.1–1.5 cm and severe if valve area < 1 cm). Aortic regurgitation was labelled as significant if it was seen in two planes, was at least 1 cm from the coaptation point of the valve leaflets, and was high velocity.^12^

## Complications of rheumatic heart disease

Following a pre-study survey of common complications presenting on our wards, a consensus was made to profile the following complications as they occurred:

• Heart failure, which was defined according to the Framingham criteria,^13^ and New York Heart Association functional status.
• Acute rheumatic fever was defined according to the 1992^14^ and 2003 WHO modified Jones criteria for diagnosis of ARF recurrence in patients with RHD.^15^
• Infective endocarditis was diagnosed according to standard criteria as previously published.^16^
• Atrial fibrillation was diagnosed using the blinded Minnesota code.^17^
• Stroke was diagnosed during history and clinical examination, and confirmed on brain computer tomography scan (CT scan).
• Pulmonary hypertension (PAH) was diagnosed based on clinical examination (findings of a loud second heart sound, murmur of tricuspid regurgitation, dilated pulmonary arteries on a chest X-ray) and confirmed using echocardiography. Doppler interrogation of tricuspid valve regurgitation was used to quantify the pulmonary arterial pressure. Pulmonary arterial systolic pressure (PASP) over 36 mmHg was defined as pulmonary hypertension.^18^

## Statistical analysis

Data were double entered and stored in EPI data version 3.0 (EpiData Association, Odense M, Denmark). Analysis was done using STATA 10.0 statistical package (Stata Corporation,College Station, TX, USA). Categorical variables were analysed using the Chi-square test, while continuous variables were analysed using the independent-samples two-tailed Student’s *t*-tests. Results are expressed as means ± standard deviation. In all statistical tests, *p* < 0.05 was regarded as significant.

## Results

**Fig. 1.**
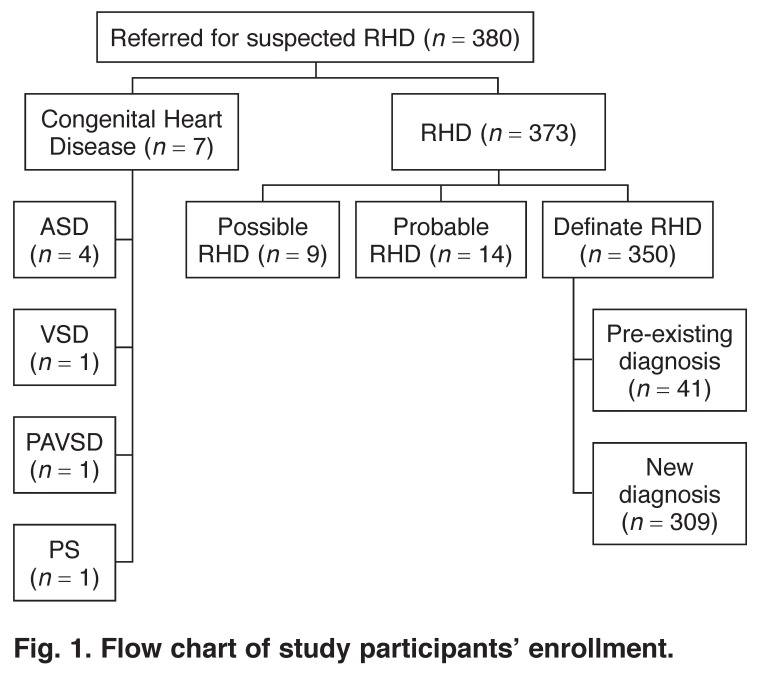
Flow chart of study participants’ enrollment.

Three-hundred and eighty patients with suspected RHD were referred for study enrollment. Congenital heart disease was found in seven patients, who were excluded from the analysis. RHD was found in 373 patients and 350 of these met the criteria for definite RHD. Of these 350 patients, 41 had a previous diagnosis of RHD and were excluded from the analysis. The remaining 309 patients, newly diagnosed as definite RHD, comprised this analysis (Fig. 1).

Demographic information for the study population can be found in Table 1. The median age in males and females was 30 years, with an interquartile range of 20. Most cases were of low socio-economic status with little or no education and unemployed.

**Table 1:** Demographic Profile Of RHD Cases

*Patient characteristics*	*n = 309 (%)*
Age (years), median (IQR)	30 (20)
Gender	
Male	113 (36.6)
Female	196 (63.5)
Education	
None	23 (7.4)
Primary	142 (45.9)
Secondary	99 (32.0)
Tertiary (college/university)	45 (14.6)
Occupation	
Peasant farmer	147 (49.1)
Civil servant	44 (14.7)
Small business owner	33 (11.4)
Student/pupil	73 (24.58)
Medication after diagnosis	
Diuretics	299 (98.4)
Benzathine penicillin	268 (97.1)
Beta-adrenergic blockers	223 (77.2)
Digoxin	212 (74.6)
Angiotensin converting enzyme inhibitors	43 (16.5)
Warfarin	48 (18)
Antiplatelates	9 (3.4)
Beta-adrenergic blockers	223 (77.2)
Calcium channel blockers	3 (1.2)
Diuretics	299 (98.4)
Ace inhibitors	43 (16.5)
Height (cm), median (IQR)	162 (12)
Weight (kg), mean (SD)	55.2 (13.8)
Pulse rate (beats/min), median (IQR)	90 (33)

IQR = inter-quartile range; SD = standard deviation.

Cardiovascular symptoms, including palpitations, fatigue, chest pain, oedema and syncope were found in 85.1% of the study enrolments. Palpitations were by far the most common complaint, being found in 84.5% of the total population, with equal distribution between males and females (Table 2).

**Table 2:** Clinical Presentation And Complications

*Complication*	*Total n (%)*	*Males n (%)*	*Females n (%)*
Total cases	309 (100)	113 (36.6)	196 (63.5)
Symptoms			
Palpitations	263 (85.1)	117 (44.4)	146 (56.6)
Fatigue	261 (84.5)	138 (52.8)	123 (47.2)
Chest pain	138 (44.7)	59 (42.7)	79 (57.3)
Oedema	112 (36.2)	36 (32.1)	76 (67.9)
Syncope	20 (6.4)	13 (65)	7 (35)
Complications			
Congestive heart failure	145 (46.9)	56 (49.5)	89 (45.4)
Acute rheumatic fever	35 (11.4)	16 (14.3)	19 (9.7)
Stroke	4 (1.3)	1 (0.9)	3 (1.5)
Clinically symptomatic peripheral embolism	0	0	0
Major bleeding	0	0	0
Infective endocarditis	14 (4.5)	7 (6.2)	7 (3.6)
Atrial fibrillation	43 (13.9)	16 (14.2)	27 (13.8)
Pulmonary hypertension	98 (32.7)	37 (32.7)	61 (31.1)

Complications were found in 49% (152/309) of the newly diagnosed RHD cases. Heart failure (46.9%) was the commonest complication, followed by pulmonary hypertension (32.7%), atrial fibrillation (13.9%), acute recurrence of rheumatic fever (11.4%), infective endocarditis (4.5%) and stroke (1.3%). (Table 2). Pulmonary hypertension was observed in about 33% of cases with no difference between male and female respondents. The mitral (70%) and aortic valves (30%) were the most affected valves in those with infective endocarditis.

Mitral regurgitation (MR) was the most common valvular lesion, followed by mixed mitral and aortic regurgitation, mitral stenosis and mixed-valve disease (Table 3). The average left ventricular end-diastolic diameter (LVEDD), average left ventricular end-systolic diameter (LVESD) and average ejection fraction (LVEF) for cases with MR was 65 mm (normal range 35–55 mm), 50 mm (normal range 25–40 mm) and 54% (normal range 40–70%), respectively, with 52% of the cases presenting with severe MR. Those with aortic regurgitation (AR) had average LVEDD, LVESD and LVEF of 58 mm, 44 mm and 56%, respectively, with 46% presenting with severe AR; 49% of the mitral stenosis cases were graded as severe (Table 3).

**Table 3:** Valve Lesions Versus Complications

	*Total n (%)*	*Mitral stenosis n (%)*	*Mitral regurgitation n (%)*	*Aortic stenosis n (%)*	*Aortic Regurgitation n (%)*
Total cases	309 (100)	104 (33.6)	280 (90.6)	8 (2.6)	128 (41.4)
Age, median (IQR)	30 (20)	33 (15)	30 (14)	30 (14)	29 (19)
Gender					
Male	113 (36.6)	31 (27.4)	102 (90.3)	4 (3.5)	45 (39.8)
Female	196 (63.5)	73 (37.2)	178 (90.8)	4 (2.0)	83 (42.4)
Complications					
Heart failure	145 (46.9)	54 (37.2)	131 (90.3)	1 (0.7)	58 (40.0)
ARF	35 (11.4)	9 (25.7)	33 (94.3)	0	10 (28.6)
Stroke	4 (1.3)	2 (50)	4 (100)	0	2 (50)
Infective endocarditis	14 (4.5)	5 (35.7)	13 (92.9)	0	6 (42.8)
Atrial fibrillation	43 (13.9)	25 (58.1)	35 (81.4)	0	14 (32.6)
Dimensions					
Mean LVEDD (mm)	58 ± 17	48 ± 9	65 ± 13	50 ± 9	58 ± 14
LVEDD > 55 mm	265 (85.7)	9 (8.7)	219 (78.2)	3 (37.5)	56 (43.8)
Mean LVESD (mm)	40 ± 11	33 ± 11	50 ± 13	37 ± 18	44 ± 15
LVESD > 45 mm	148 (47.8)	5 (4.8)	193 (68.9)	3 (37.5)	29 (22.5)
Mean LAD (mm)	53	63	55	45	47
Mean LVEF (%)	59.7	67	54	59.5	56.3
Systolic dysfunction	101.9 (33)	9 (9)	126 (45)	3 (12)	38 (30)
Severity of valve lesion (%)	mild: moderate: severe	23:28:49	20:28:52	24:53:23	13:41:46

An analysis of factors associated with congestive heart failure was performed. Atrial fibrillation was the commonest complication associated with congestive heart failure (81.4% CHF vs 18.6% no CHF, *p* < 0.05), followed by acute rheumatic fever recurrence (71.4% CHF vs 28.6% no CHF, *p* = 0.002), and infective endocarditis (78.6% CHF vs 21.4% no CHF, *p* = 0.015) (Table 4).

**Table 4:** Bivariate Analysis Of Heart Failure Predictors

	*Congestive heart failure*		
	*Yes, n (%)*	*No, n (%)*	p*-value*
Male	56 (49.6)	57 (50.4 )	
Female	89 (45.4)	107 (54.6)	0.225
Systolic blood pressure, mean ± SD	117 ± 1.76	124 ± 1.82	0.006
Diastolic blood pressure, median (IQR)	70 (60–82)	71 (63–84)	0.614
Other complications of RHD			
Acute rheumatic fever	25 (71.4)	10 (28.6)	0.002
Stroke	1 (50)	2 (50)	0.906
Infective endocarditis	11 (78.6)	3 (21.4)	0.015
Atrial fibrillation	35 (81.4)	8 (18.6)	< 0.05

In addition, 50% of cases with stroke presented in atrial fibrillation. The rest had mitral stenosis in sinus rhythm; 35 cases presented with recurrence of ARF. Average ASO titre and C-reactive protein levels were 526.7 mg/l (normal range 0.00–200.0) and 97.4I U/ml (normal range 0.0–5.0), respectively. In addition to the carditis, joint pain and chorea were the only extracardiac manifestations of acute rheumatic fever observed. All cases of recurrence of ARF presented in heart failure.

## Discussion

Rheumatic heart disease remains a common cause of heart failure in developing countries and ranks among systemic hypertension and idiopathic dilated cardiomyopathy as the leading causes of heart failure in these nations.^19^,^20^ Rheumatic heart disease is one of the most common cardiac diagnoses seen in Uganda.^21^ Among our cohort of patients newly presenting with RHD, almost 90% reported cardiovascular symptoms and almost 50% had already developed related complications.

This is the first study to profile the presentation of rheumatic heart disease in Uganda and highlights the tendency for late disease presentation and high rates of associated complications.

Demographic information obtained from this study agrees with previous studies that RHD is a disease of low socio-economic status and low educational level, as the majority of the respondents had either primary-level or no education.^22^,^23^ Our data continue to highlight that RHD is a disease of poverty, low productivity, poor quality of life and early mortality.^19^,^20^

In our cohort, mitral regurgitation was the commonest valve lesion, with the majority presenting in heart failure, and about half presenting with severe mitral valve regurgitation, with average LVEDD, LVESD and LVEF of 65 mm, 50 mm and 54%, respectively This is severe valve disease and recent studies have showed that an average LVESD of 50 mm or more is associated with rapid decline in cardiac function.^24^ In addition, it has been shown that delayed surgery is associated with a poor postoperative prognosis.^24^,^25^

Furthermore, Sliwa *et al.* in a study of the clinical characteristics of newly diagnosed RHD as seen in the Heart of Soweto study, South Africa,^5^ found the prevalence of newly diagnosed RHD to be 36% among patients diagnosed with any valvular heart disease, out of an overall total of 4 005 cases. Mitral regurgitation was the commonest valve lesion. Only 25% of cases had average LVDD > 55 mm, lower than that in our study where 52% of cases with mitral regurgitation had average LVEDD > 55 mm. This indicates that the cases in their study probably presented or were picked out earlier.

Cardiovascular symptoms typically manifest when valvular regurgitation or stenosis (regardless of valve) becomes severe.^26^ This was demonstrated in our population, where 52% of patients had severe mitral regurgitation with average LVESD > 55 mm, and 80% of these patients reported symptoms.

Among our cohort, the complication rate at presentation was almost 50%. This is slightly higher than reported by Sani *et al.*, who studied the prevalence and pattern of RHD in the Nigerian savanah. In their echocardiography-based study, 32% had left ventricular dysfunction, among other complications, at the time of initial diagnosis.^6^ This is slightly lower than our reported rate of 49%.

In our population, pulmonary hypertension was the second most common complication, affecting almost one-third of patients at the time of presentation. Males and females were affected equally. Pulmonary hypertension commonly develops as a complication of mitral and aortic valve disease.^27^ Development of pulmonary hypertension decreases quality of life and shortens life expectancy.^27^,^28^

Recurrent ARF was found in 11% of patients at the time of the initial diagnosis. Most of these cases did not fulfill the classical Jones criteria.^14^,^29^ Joint pains, chorea and evidence of Group A streptococcal infection, as shown by raised ASO titres were the only criteria met. These findings support the use of the 2003 WHO modification of the Jones criteria^29^ for the diagnosis of recurrence of ARF in established RHD.

The tendency of patients to present during an episode of recurrent ARF has been previously described. Ravisha and colleagues reported data on 550 cases of newly diagnosed RHD in India.^19^ In their study, almost 40% of newly diagnosed patients were presenting with recurrence of ARF, and the rate of heart failure in these patients was 36%. We report that 100% of those presenting with ARF met the criteria for heart failure, which is most likely what brought them to our attention.

It is not completely understood whether the heart failure that occurs in ARF is due to myocarditis or severe valve damage. Essop *et al.*^30^ suggested that the heart failure observed during ARF is probably secondary to volume overload from the valve lesion, not primary myocardial dysfunction. Our data suggest that myocardial dysfunction contributes to the heart failure seen, as patients with ARF had decreased left ventricular ejection fraction (mean 44%). Whether the myocardial dysfunction is attributable to acute myocarditis or secondary to acute on chronic volume overload remains unknown. In the future, myocardial biopsy might provide this answer and help direct therapy.

Silwa and collegues^5^ report a different experience with recurrent ARF. In their study, there were no cases of ARF at the time of the initial RHD diagnosis. This difference is likely attributable to the notifiable nature of ARF in South Africa. Among countries in sub-Saharan Africa, active surveillance for new ARF cases is unique to South Africa.

As we are reporting only new diagnoses, no patient in our cohort was receiving benzathine penicillin. Benzathine penicillin prophylaxis reduces recurrence of ARF to less than 20% in those who achieve at least 80% adherence.^8^ Two to three weekly benzathine penicillin injections are now thought to offer better protection than the older recommendation of injections every four weeks.^31^,^32^

Atrial fibrillation was found in almost 14% of our patients, and was the third most common complication. Worldwide, atrial fibrillation is the most common sustained arrhythmia and is associated with complications such as heart failure, stroke and other embolic phenomena.^33^ In our cohort, atrial fibrillation had the strongest association with heart failure; 81.4% of our patients with atrial fibrillation had heart failure. This was not surprising given that the average left atrial diameter was 5.5 cm in the cases with atrial fibrillation. Previous studies have showed that patients with dilated atria over 5.0 cm are less likely to remain in sinus rhythm even after attempted cardioversion or ablation.^34^ Patients with atrial fibrillation are also at increased risk of cardio-embolic phenomena, secondary to stasis of blood and clot formation.^35^,^36^

In the present study, four patients presented with stroke, all of whom had concurrent atrial fibrillation. No patients were on anticoagulant medication at presentation. The best strategy for medical management of this population in the developing world is debatable. Fearing side effects, clinicians often hesitate to prescribe anticoagulation in settings where reliable dosing and monitoring of INR levels is difficult. Yet, this can have dire consequences for the patient.^37^,^38^ Evidence is clear from developed nations that patients with atrial fibrillation have decreased stroke when properly anticoagulated.^39^,^40^ It is our practice to concomitantly begin low-molecular weight heparin and coumadin at the time of anti-arrythmia initiation (for either rate or rhythm control).^41^

The main limitation to this study was that it was conducted at the national referral hospital where severe cases are typically referred for treatment. This may have under-represented the number of patients with milder forms of the disease, who are seen at lower levels of the healthcare system.

## Conclusion

We describe the first report of RHD presentation in Uganda. We have compiled a profile of symptoms and complications in 309 patients, including symptoms and complications at the time of presentation. Almost all (88%) patients were symptomatic, and half had already developed complications from RHD. Patients presented late in the disease course, suggesting there may be opportunity for earlier intervention.

In 2012, the World Heart Federation published the first evidence-based guidelines for echocardiographic screening in RHD.^42^ Implementation of a screening programme using these guidelines may be an effective way to detected cases early, when patients have the most to gain from secondary prophylaxis.

We also noted that recurrence of acute rheumatic fever was high in our study. This underscores the urgent need to improve patients’ and healthcare providers’ knowledge of the diagnosis and treatment of streptococcal infections, as well as delivery and adherence to secondary prophylaxis. It is clear that there is much work to be done to prevent RHD and to ensure patients who develop RHD are diagnosed before symptoms and complications develop. Raising awareness of the burden of RHD, as well as the development of local guidelines for screening, diagnosis and management could begin to lessen the devastation of this all too-common disease.
